# 1-Butyl-3-methylimidazolium Mandelate: A Multifunctional Ionic Liquid with Enhanced Hydrogen Bonding, Thermal Stability, Antimicrobial Activity, and Extraction Capability

**DOI:** 10.3390/molecules30244824

**Published:** 2025-12-18

**Authors:** Nikolett Cakó Bagány, Eleonora Čapelja, Sanja Belić, Dajana Lazarević, Jelena Jovanović, Tatjana Trtić-Petrović, Slobodan Gadžurić

**Affiliations:** 1 Faculty of Science, University of Novi Sad, Trg Dositeja Obradovića 3, 21000 Novi Sad, Serbia; nikolet.cakobaganj@dh.uns.ac.rs (N.C.B.); eleonora.capelja@dbe.uns.ac.rs (E.Č.); sanja.belic@dh.uns.ac.rs (S.B.); 2 Laboratory of Physics, Vinča Institute of Nuclear Sciences–National Institute of the Republic of Serbia, University of Belgrade, Mike Petrovića Alasa 12-14, 11001 Belgrade, Serbia; dajana.lazarevic@vinca.rs (D.L.); jelena.jovanovic@vinca.rs (J.J.); ttrtic@vinca.rs (T.T.-P.)

**Keywords:** ionic liquids, mandelate, extraction, ABS, viscosity, aggregation behavior, density, antimicrobial activity

## Abstract

Designing ionic liquids (ILs) where a single functional group orchestrates a suite of enhanced properties remains a key challenge in materials science. Here, we introduce 1-butyl-3-methylimidazolium mandelate, [Bmim][Man], a novel IL where the hydroxyl group on the mandelate anion simultaneously enhances hydrogen bonding, thermal stability, antimicrobial activity, and extraction selectivity. The structure-property relationships of [Bmim][Man] were investigated through measurements of density, viscosity, and conductivity and were compared with analogous ILs. The presence of the hydroxyl group on the mandelate anion resulted in the highest density and viscosity among the series, attributed to strong hydrogen bonding and efficient ion packing. Notably, [Bmim][Man] exhibited a high molar conductivity that decouples from its high viscosity, suggesting an unusual degree of ion dissociation facilitated by the hydroxyl group. Thermogravimetric analysis revealed superior thermal stability. Furthermore, the investigated ionic liquid demonstrated a low critical aggregation concentration (CAC = 0.01982 mol·dm^−3^) in water, indicating a strong propensity for self-aggregation. [Bmim][Man] showed synergistic, enhanced antibacterial activity against *E. coli* and *P. aeruginosa*. Finally, the functional utility of this designed liquid was demonstrated in separation science, where [Bmim][Man]-based aqueous biphasic systems showed selective extraction capabilities for transition metals, a process driven by the same hydrogen-bonding and coordination interactions that define its bulk properties. These findings establish [Bmim][Man] as a promising multifunctional material where the mandelate anion concurrently dictates liquid microstructure, thermal resilience, antimicrobial performance, and application in extraction.

## 1. Introduction

In recent years, ionic liquids (ILs) have evolved from being considered merely alternative solvents to becoming multifunctional materials with tunable physicochemical and biological properties [1,2,3,4]. While the cation often determines the overall structural framework of an IL, it is now widely recognized that the anion plays a decisive role in controlling specific interactions within the liquid phase, its organization in solution, and its performance in applications [5]. For this reason, current research interest has shifted toward designing anions with targeted functionalities that can influence both molecular transport and biological activity [6,7]. Our earlier studies involving the ionic liquids 1-butyl-3-methylimidazolium benzoate, [Bmim][Ben], 1-butyl-3-methylimidazolium phenylacetate, [Bmim][Phe], and 1-butyl-3-methylimidazolium 4-methoxyphenylacetate, [Bmim][CH_3_OPhe], highlighted the role of the aromatic anion and the importance of π-interactions and hydrophobic effects in shaping viscosity and conductivity trends. These observations are in line with recent literature on carboxylate-based ionic liquids [8]. However, these systems could not establish strong intermolecular hydrogen-bonding networks. The next logical step is to explore an aromatic anion capable of stronger, directional intermolecular interactions that could reshape ion pairing and microstructural organization in both the pure IL and its aqueous solutions [9,10]. The introduction of a hydroxyl substituent on the aromatic ring offers a new level of complexity: such groups can generate directional interactions, alter solvation behavior, and potentially promote micro-structuring both in the pure IL and in aqueous media. Mandelate fulfills these criteria precisely. In contrast to previously investigated anions, mandelate is attractive because it combines three structural features in a single anion: an aromatic ring, a hydrogen-bond-donating hydroxyl group, and a chiral center, which may introduce asymmetry into the ionic network and potentially affect packing, molecular mobility, and aggregation behavior. Additionally, mandelic acid and its derivatives are known for their strong antimicrobial effects, already in use in medicine and cosmetics, making mandelate-based ionic liquids good candidates for applications that need both good physicochemical properties and biological activity.

Mandelic acid is an α-hydroxy acid with low toxicity and well-documented antibacterial activity against microorganisms like *Staphylococcus aureus*, *Escherichia coli*, *Proteus* sp., and *Pseudomonas* sp., acting through membrane disruption, induction of osmotic and oxidative stress, and denaturation of surface proteins by hydrogen bonding [11,12]. The coexistence of aromaticity, hydrogen-bonding capability, and biological activity makes mandelate a highly relevant anion for designing bioactive ionic liquids with tunable microstructural organization and enhanced antimicrobial performance.

Furthermore, the distinct solvation microenvironment created by the mandelate anion, characterized by its hydrophobicity, hydrogen-bonding capacity, and π-electron density, suggests potential for application in separation processes. Aqueous Biphasic Systems (ABS) formed by ILs are effective platforms for the extraction and partitioning of valuable biomolecules and metal ions [13,14,15,16]. The specific interactions afforded by the mandelate anion, such as coordination with metal cations and hydrogen bonding with organic solutes, were therefore investigated to evaluate the utility of [Bmim][Man] in such extraction processes [17,18], further demonstrating its multifunctional character. Accordingly, the present work focuses on the ionic liquid 1-butyl-3-methylimidazolium mandelate, aiming to investigate how the introduction of a hydroxyl-functionalized aromatic anion affects its liquid structure and macroscopic properties.

To the best of our knowledge, this is the first comprehensive report on the synthesis, physicochemical characterization, and multifunctional application of 1-butyl-3-methylimidazolium mandelate. The study includes measurements of density, viscosity, and conductivity for the pure IL, together with viscosity evaluation in aqueous solutions, thermal analysis, and assessment of antimicrobial activity. Herein, we report that [Bmim][Man] indeed functions as a multifunctional material, exhibiting enhanced hydrogen bonding, superior thermal stability, synergistic antimicrobial activity, and effective extraction capabilities for transition metals.

## 2. Results and Discussion

### 2.1. Experimental Density, Viscosity, and Conductivity of Pure Ionic Liquid

**Figure 1:**
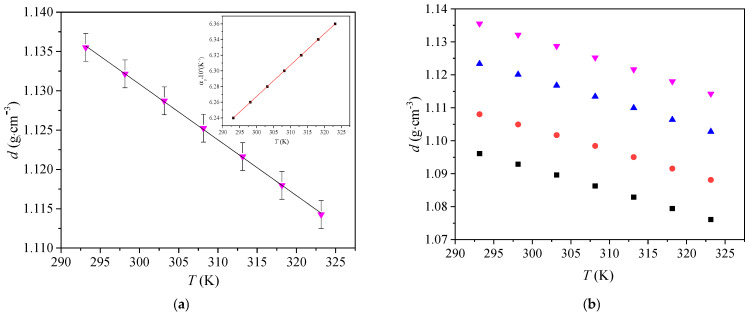
(**a**) Variation in [Bmim][Man] density with temperature, inset: Variation in thermal expansion coefficients *α*_p_ of [Bmim][Man] with temperature (**b**) Comparison of the densities of [Bmim]^+^ ionic liquids with different anions: ■ [Bmim][Phe]; ● [Bmim][Ben]; ▲ [Bmim][CH_3_OPhe] and ▼ [Bmim][Man].

**Figure 2:**
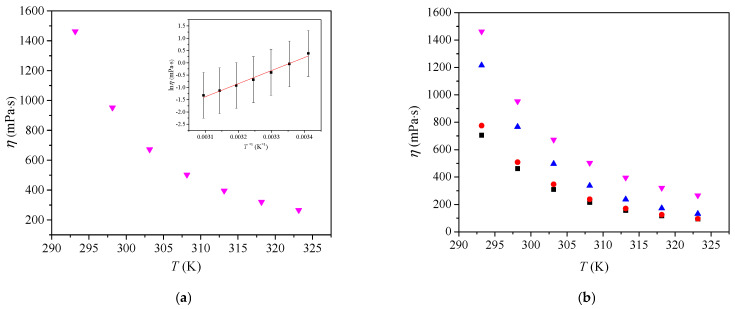
(**a**) Variation in [Bmim][Man] viscosity with temperature, inset: Variation in ln *η* with *T^−^*^1^ (**b**) Comparison of the viscosities of [Bmim]^+^ ionic liquids with different anions: ■ [Bmim][Phe]; ● [Bmim][Ben]; ▲ [Bmim][CH_3_OPhe] and ▼ [Bmim][Man].

**Figure 3:**
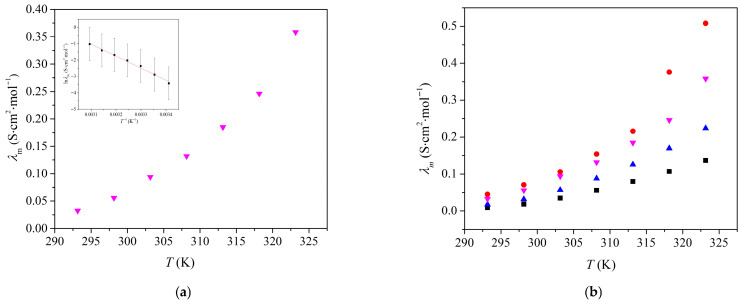
(**a**) Variation in [Bmim][Man] molar conductivity with temperature, inset: Variation in ln *λ_m_* with *T*^−1^ (**b**) Comparison of the molar conductivities of Bmim ionic liquids with different anions: ■ [Bmim][Phe]; ● [Bmim][Ben]; ▲ [Bmim][CH_3_OPhe] and ▼ [Bmim][Man].

The structure-property relationships of [Bmim][Man] were investigated through measurements of density, viscosity, and conductivity and were compared with analogous ILs-[Bmim][Ben], [Bmim][Phe], and [Bmim][CH_3_OPhe]. Densities (*d*), viscosities (*ƞ*), and electrical conductivities (*κ*) of pure [Bmim][Man] were measured in the temperature range from *T* = (293.15 to 323.15) K, at atmospheric pressure (*p* = 0.1 MPa). The results are shown in Table S2 and illustrated in Figure 1, Figure 2 and Figure 3.

From the measured density values, the thermal expansion coefficient, *α*_p,_ of pure ionic liquids is calculated using the Equation (1):

(1) $$\alpha_{p}=-\frac{1}{d}{\left(\frac{𝜕d}{𝜕T}\right)}_{p,m}$$

As shown in Figure 1a, the density of pure [Bmim][Man] decreases linearly with increasing temperature, which is typical behavior for most ionic liquids [1,2]. Correspondingly, *α*_p_ increases with increasing temperature. Figure 1b shows a comparison of the densities of ionic liquids containing various anions (data for other ionic liquids are taken from our previous research) [8]. Among the compounds studied, [Bmim][Man] demonstrates the highest density across the entire temperature range. This phenomenon can be attributed to the presence of the hydroxyl group in the mandelate anion, which facilitates strong hydrogen bonding [9]. This results in tighter ion pairing and more efficient molecular packing.

In comparison, the methoxy-substituted ionic liquid [Bmim][CH_3_OPhe] also shows relatively high density due to the polar character of the –OCH_3_ group; however, it does not promote hydrogen bonding to the same extent as the hydroxyl group. [Bmim][Ben] and [Bmim][Phe], which lack polar substituents on the aromatic ring, show lower densities, consistent with weaker cation-anion interactions and more free volume in the liquid structure [8].

These results indicate that functional groups on the anion significantly influence ionic liquid density, with hydrogen-bond donors, such as –OH, contributing more to dense packing than alkyl or ether substituents.

The variation in viscosity with temperature was fitted using the logarithmic form of the Arrhenius equation:

(2) $$\ln\eta =-\frac{E_{a_{1}}}{RT}+\ln C$$

where *C* is the pre-exponential coefficient, *E*_a1_ is the activation energy of viscous flow (*E*_a1_ = 639.5 kJ·mol^−1^), and *R* is the universal gas constant.

As shown in Figure 2a, the viscosity of [Bmim][Man] decreases with temperature increase. A similar trend was observed for viscosity as for density (Figure 2b): [Bmim][Man] exhibited the highest viscosity among the studied ionic liquids, followed by [Bmim][CH_3_OPhe], [Bmim][Ben], and [Bmim][Phe] reported in [8]. This behavior is consistent with the nature of the anion and its ability to form strong interactions. The hydroxyl group in the mandelate anion contributes to stronger hydrogen bonding, which restricts ion mobility and increases resistance to flow, resulting in higher viscosity. In contrast, ionic liquids with less polar or non-polar substituents, such as benzoate or phenylacetate, demonstrate lower viscosities due to weaker intermolecular interactions and greater free volume. The methoxy group in [Bmim][CH_3_OPhe] slightly increases viscosity compared to non-functionalized anions, but its effect is less pronounced than that of the hydroxyl group. These results show that the presence and nature of functional groups on the anion significantly affect both the density and viscosity of ionic liquids, primarily through their influence on intermolecular interactions and structural organization.

The variation in molar conductivity given in Table S2 with temperature is shown in Figure 3. The obtained dependence was fitted using the logarithmic form of the Arrhenius equation:

(3) $$\ln\lambda_{m}=-\frac{E_{a2}}{RT}+\ln C$$

where *E*_a2_ is the conductivity activation energy (*E*_a2_ = 878.0 kJ·mol^−1^). The obtained activation energies *E*_a1_ and *E*_a2_ are higher than those typically reported for non-functionalized ILs, which we attribute to the pronounced hydrogen-bonding network and tight ion packing facilitated by the hydroxyl group on the mandelate anion.

As shown in Figure 3a, the molar conductivity of [Bmim][Man] increases with temperature, which is expected due to the enhanced ion mobility at higher temperatures. Among the ionic liquids studied (Figure 3b), [Bmim][Ben] exhibits the highest molar conductivity, followed by [Bmim][Man], [Bmim][CH_3_OPhe], and [Bmim][Phe] [8]. Interestingly, despite having a higher viscosity, [Bmim][Man] demonstrates greater molar conductivity than both [Bmim][CH_3_OPhe] and [Bmim][Phe]. This observation can be attributed to the presence of the hydroxyl group in the mandelate anion, which facilitates stronger ion-dipole and hydrogen bonding interactions. These interactions can enhance ion dissociation and increase the number of free charge carriers, thereby compensating for the reduced mobility caused by the higher viscosity.

### 2.2. Antimicrobial Activity of [Bmim][Man]

**Figure 4:**
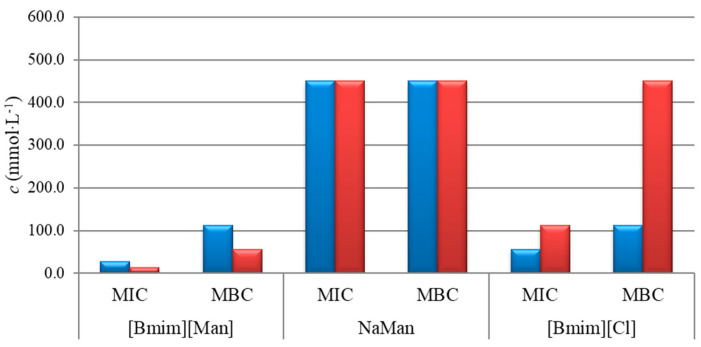
Antibacterial activity of the investigated ionic liquid, [Bmim][Man] and its individual anionic/cationic components against *E. coli* (blue) and *P. aeruginosa* (red).

**Figure 5:**
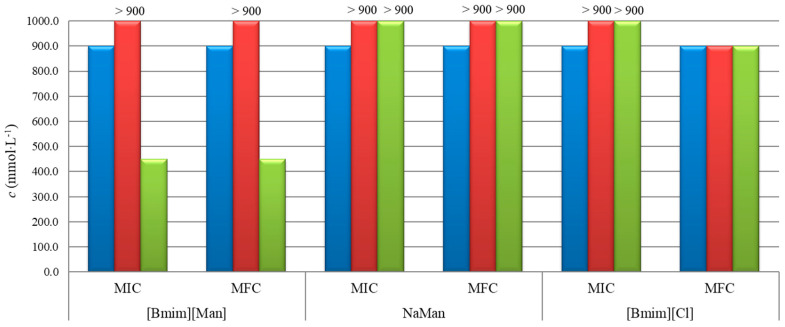
Antifungal activity of tested IL, [Bmim][Man] and its individual anionic/cationic components against *P. verrucosum* (blue), *A. flavus* (red), and *A. parasiticus* (green).

The results of the minimum inhibitory concentration (MIC), minimum bactericidal concentration (MBC), and minimum fungicidal concentration (MFC) are summarized in Table 1 and illustrated in Figure 4 and Figure 5.

The results presented in Figure 4 indicate that the ionic liquid [Bmim][Man] exhibits markedly higher antibacterial activity compared to its individual ionic components, NaMan (anion) and [Bmim][Cl] (cation). Both MIC and MBC values for [Bmim][Man] are significantly lower, suggesting a synergistic interaction between the cationic and anionic parts within the ionic liquid. This synergistic effect is consistent with mechanisms reported for imidazolium-based ILs (membrane disruption) and mandelate derivatives (induction of osmotic/oxidative stress) [11,12], though direct mechanistic confirmation would require further dedicated studies.

The results demonstrate (Figure 5) that the ionic liquid, [Bmim][Man], exhibits moderate antifungal activity against *A. parasiticus*, with MIC and MFC values around 450 mmol·L^−1^. In contrast, no significant antifungal effect was observed against *P. verrucosum* and *A. flavus*, as their MIC and MFC values exceeded 900 mmol·L^−1^. Both individual ionic components, NaMan and [Bmim]Cl, showed no notable activity against any of the tested fungi, with MIC and MFC values exceeding the highest concentration tested (900 mmol·L^−1^).

Compared to analogous carboxylate-based ILs such as [Bmim][Ben] and [Bmim][Phe] [8], [Bmim][Man] exhibits markedly lower MIC values against *E. coli* and *P. aeruginosa*, highlighting the beneficial role of the hydroxyl substituent in enhancing antimicrobial potency. This places [Bmim][Man] among the more active ILs designed for dual-functionality applications.

### 2.3. Viscosities of [Bmim][Man] Aqueous Solutions

**Figure 6:**
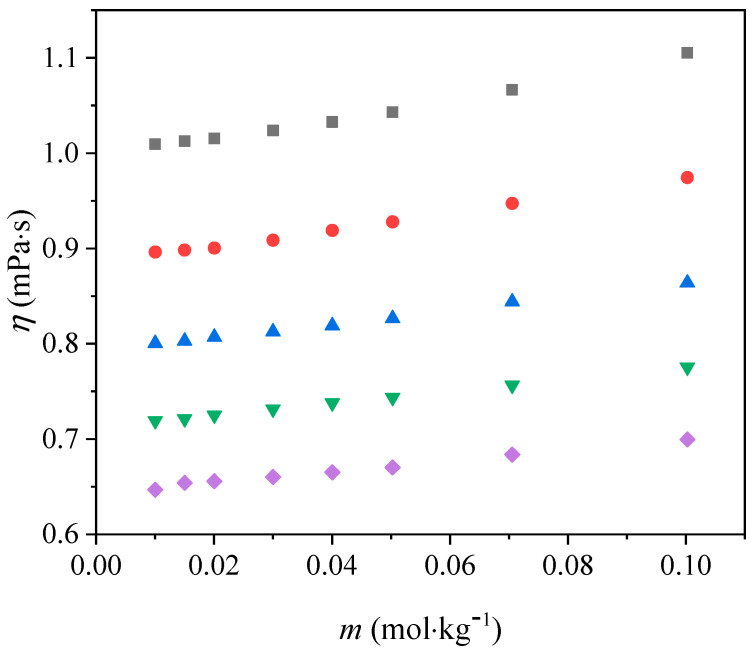
Variation in viscosity with molality for [Bmim][Man] aqueous solutions at different temperatures: ■ 293.15 K; ● 298.15 K; ▲ 303.15 K; ▼ 308.15 K and ♦ 313.15 K.

The viscosities of [Bmim][Man] aqueous solutions were measured in the molality range from *m* = (0.01001 to 0.10025) mol·kg^−1^, and across the temperature range from *T* = (293.15 to 313.15) K. Results are presented in Table S3 and plotted in Figure 6.

From the values tabulated in Table S3 and plotted in Figure 6, it can be observed that the viscosity of aqueous solutions increases with increasing molality. As the molality rises, the number of solute particles in the solution also increases, leading to stronger interactions between ions and water molecules. These enhanced ion-solvent and ion-ion interactions hinder the mobility of the molecules, resulting in higher viscosity values.

On the other hand, as the temperature increases, the viscosity of the aqueous solutions decreases. This occurs because higher temperature provides more thermal energy to the molecules, which weakens the intermolecular forces and enhances molecular motion. Consequently, the resistance to flow (viscosity) is reduced.

In summary, viscosity rises with increasing concentration due to stronger interparticle interactions, while it decreases with temperature because thermal motion overcomes these interactions.

The viscosity data were analyzed using both the Jones-Dole and the modified Jones-Dole equations:

(4) $$\eta_{r}=\frac{\eta }{\eta_{0}}=1+A\cdot \sqrt{c}+B\cdot c$$

(5) $$\;\eta_{r}=1+B\cdot c$$

The coefficients *A* and *B* describe ion-ion and ion-solvent interactions, respectively. The obtained results revealed a clear breakpoint in the concentration dependence of relative viscosity, indicating the onset of self-aggregation due to the predominance of solute-solute over solute-solvent interactions. Beyond this point, the *A* coefficient can be neglected, and the modified form of the Jones-Dole equation was applied. Since an identical procedure was employed in our previous work, only a brief description is provided here, while the detailed methodology can be found in the earlier publication [19].

The critical aggregation concentration was determined using the Vand equation, and the results agree with the data obtained by other methods.

(6) ln(ηη0)=2.5·ϕ1−K·ϕ

where *ϕ* is the volume fraction, *K* is termed a particle interaction constant. Converting to decade logarithms and rearranging, the equation becomes:

(7) 2.5·ϕln(ηη0)=2.303−2.303K·ϕ

By substituting *ϕ* = *c*·*V*_e_ (*V*_e_ is the effective volume) and rearranging, the modified expression is obtained:

(8) $$\;\;\;\;\;\;\;\;\frac{c}{log\left(\frac{\eta }{\eta_{0}}\right)}=\frac{2.303}{2.5\cdot V_{e}}-\frac{2.303\cdot K\cdot c}{2.5}$$

**Figure 7:**
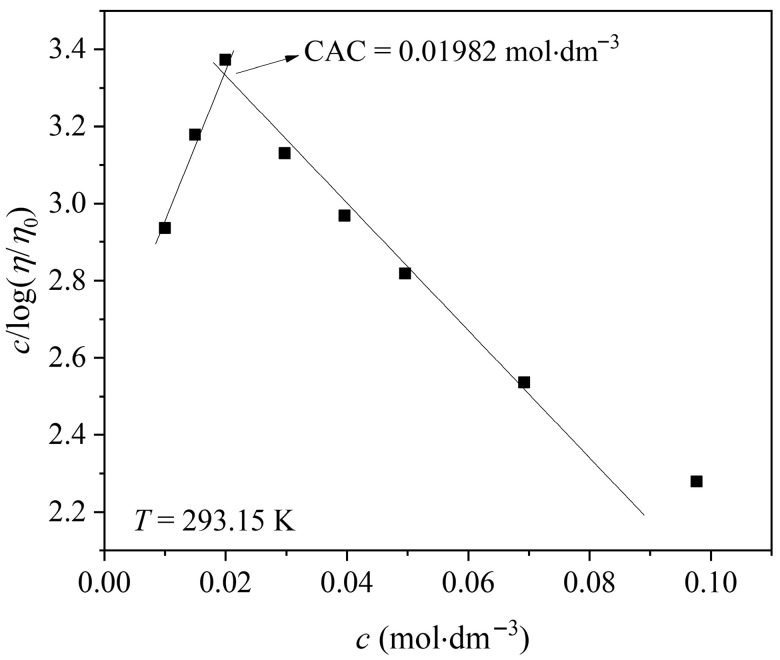
Variation in c/log(*η*/*η*_o_) with concentration of [Bmim][Man] in aqueous solution at *T* = 293.15 K.

The *c*/log*(η/η_0_)* plot for [Bmim][Man] in Figure 7 revealed a CAC value of 0.01982 mol·dm^−3^, which is notably lower than those reported previously for [Bmim][Ben] (0.02967 mol·dm^−3^), [Bmim][Phe] (0.03040 mol·dm^−3^), and [Bmim][CH_3_OPhe] (0.02321 mol·dm^−3^) [19]. This clearly indicates a stronger tendency toward self-aggregation for [Bmim][Man] in aqueous solution. The observed behavior can be attributed to the mandelate anion, which possesses both an aromatic ring and a hydroxyl group. The aromatic moiety facilitates π-π stacking interactions with the imidazolium cation, while the hydroxyl and carboxylate groups enable extensive hydrogen bonding with water molecules and other ions. These synergistic interactions promote the formation of aggregates at lower concentrations. Beyond the CAC, the viscosity increase becomes less pronounced, consistent with the transition from individual ion-solvent interactions to inter-aggregate interactions. As the ions associate into larger aggregates, the number of free, mobile ions decreases, causing changes in the viscosity and flow behavior of the solution.

Overall, these results demonstrate that [Bmim][Man] exhibits the strongest self-aggregation propensity among the studied ILs [19], owing to the combined effects of π-π stacking and hydrogen bonding. This finding further emphasizes the key role of anion structure in determining the aggregation and viscosity behavior of aqueous ionic liquid systems.

### 2.4. Thermogravimetric Measurements

**Figure 8:**
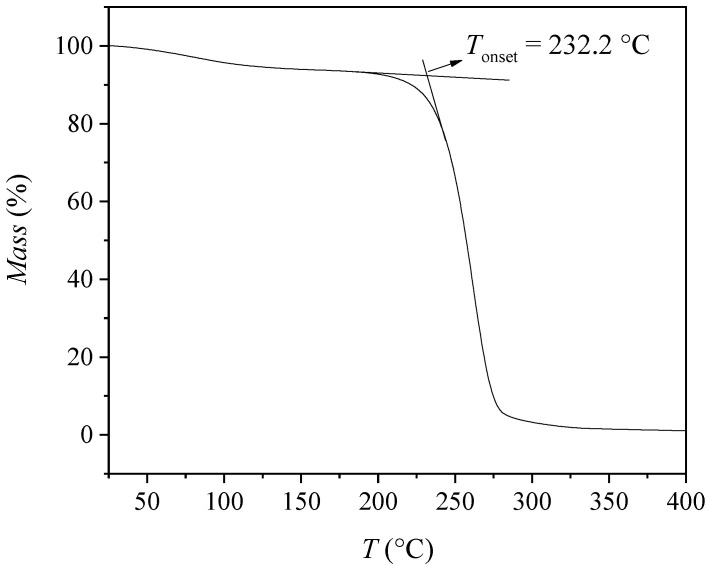
TGA curve of [Bmim][Man] from 25 to 400 °C.

The effect of the –OH functional group on the thermal stability of the pure [Bmim]-based ionic liquid was evaluated by thermogravimetric analysis (TGA) (Figure 8). In the present study, [Bmim][Man] was analyzed for thermal stability for the first time, showing an onset decomposition temperature of *T*_onset_ = 232.2 °C, which is higher than those of all previously studied ILs. The enhanced thermal robustness of [Bmim][Man] can be attributed to the mandelate anion, where the aromatic ring and hydroxyl group may provide additional stabilization through a combination of electron delocalization and intramolecular hydrogen bonding [19], making the anion less prone to early degradation under heat.

For comparison, [Bmim][CH_3_OPhe], containing an electron-donating methoxy group, showed the lowest thermal stability with *T*_onset_ = 215.9 °C, while [Bmim][Ben], with the unsubstituted benzoate anion, exhibited *T*_onset_ = 226.9 °C [17]. These differences illustrate the influence of anion substituents on thermal behavior: electron-donating groups tend to decrease stability, whereas aromatic or unsubstituted anions can enhance it. Overall, [Bmim][Man] emerges as the most thermally stable IL in this series, highlighting the significant role of anion structure in determining IL thermal properties.

### 2.5. Extraction of the Studied Metal Ions Using ABS Based on [Bmim][Man]

**Figure 9:**
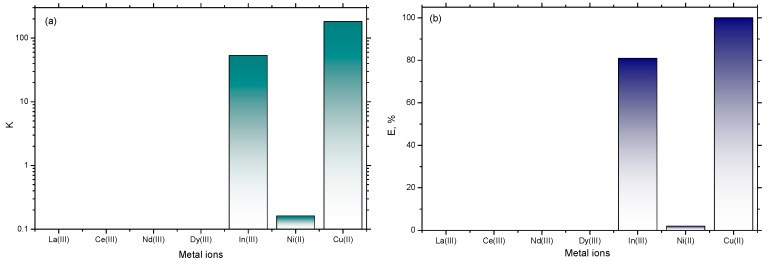
(**a**) Partitioning coefficients, and (**b**) extraction efficiency of the targeted metal ions in the ternary system {[Bmim][Man] + K_3_PO_4_ + H_2_O} at 296 K.

The partitioning of the target metal ions in the ternary {[Bmim][Man] + K_3_PO_4_ + H_2_O} system was quantitatively evaluated. The partition coefficient (*K*) and extraction efficiency (%E) for each element are summarized in Figure 9.

The data reveals a clear trend in extraction performance. The most efficient extraction was obtained for Cu^2+^ and In^3+^, with partition coefficients of 182.2 and 53.5, respectively (Figure 9a), and extraction efficiencies of 99% and 81%, respectively (Figure 9b). On the contrary, the partition coefficient of Ni^2+^ was below 1, and no significant partitioning of the investigated lanthanides into the IL-rich phase was observed.

This selective behavior can be attributed to the specific interactions between the metal cations and the functional groups on the mandelate anion. The high extraction efficiency for Cu^2+^ is likely due to its well-known strong coordination affinity with both carboxylate groups (from the mandelate) and nitrogen-containing aromatic rings (from the imidazolium cation), forming stable complexes that are highly soluble in the IL-rich phase. The superior performance of In^3+^ can also be explained by its higher complexation stability with the donor atoms present in the system. This demonstrates that the [Bmim][Man]-based ABS is not only effective for extraction but also highly selective, a key advantage for separation processes. The high selectivity of the studied ionic liquid for Cu^2+^ and In^3+^ in the presence of Ni^2+^ and the lanthanides is highly relevant for hydrometallurgy (e.g., recovering copper from nickel-containing leachates), suggesting a high potential of [Bmim][Man] for their separation and exceeding thus, our preliminarily obtained results for [Bmim][Ben] and [Bmim][Phe] based ABS under similar conditions.

Notably, the system showed a pronounced selectivity, as no significant extraction was observed for the tested Rare Earth Elements (REEs) under these conditions (Figure 9). This result aligns with the hard-soft acid-base (HSAB) principle. REEs are hard Lewis acids and are strongly complexed by the extremely hard phosphate anions in the salt-rich bottom phase. The mandelate anion, with its mixed hard-soft character, cannot effectively compete for these hard cations. In contrast, the successfully extracted transition metals, particularly the borderline/soft acids (like Cu^2+^), have a higher affinity for the coordination environment offered by the imidazolium cation and the mandelate anion. Thus, the hard REEs (e.g., La^3+^, Ce^3+^) are strongly solvated by the hard phosphate anions in the salt-rich phase, while the borderline acid Cu^2+^ favorably coordinates with the softer nitrogen of the imidazolium ring and the carboxylate oxygen of the mandelate anion, partitioning into the IL-rich phase. This inherent selectivity highlights the potential of [Bmim][Man]-based ABS for targeted separations, such as recovering valuable transition metals from complex mixtures that may also contain REEs, underscoring the advantage of the hydroxyl-functionalized mandelate anion in coordinating borderline/soft metal ions.

## 3. Materials and Methods

### 3.1. Synthesis of 1-Butyl-3-methylimidazolium mandelate

**Figure 10:**
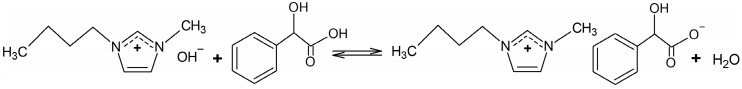
Schematic representation of [Bmim][Man] synthesis.

In the first step of the synthesis (Figure 10), 1-butyl-3-methylimidazolium chloride ([Bmim][Cl]) was dissolved in water and treated with a commercially available anion exchange resin (Amberlite IRN78) to obtain 1-butyl-3-methylimidazolium hydroxide ([Bmim][OH]). The ion exchange continued until the test for chloride ions was negative. The resulting aqueous [Bmim][OH] solution was used for titration.

For the synthesis of an ionic liquid with a carboxylate anion, a potentiometric titration method was employed. A weighed amount of the corresponding acid was dissolved in methanol and titrated with the aqueous [Bmim][OH] solution while monitoring pH. The titration curves were used to determine the equivalence point from the second derivative, which was found to be at pH = 11.41 for [Bmim][Man]. After titration, the appropriate amount of acid was added to adjust the pH to the desired value.

The solvents (water and methanol) were removed by rotary evaporation under vacuum at 343.15 K for 60 min, followed by further drying using a vacuum pump and heating until constant mass was reached.

The detailed synthesis procedure has been published in our previous work [20], and the NMR and FTIR spectra are provided in the Supplementary Material (Figures S1 and S2) of the present study. The water content was determined by the Karl Fischer titration using the 831 Karl Fischer coulometer. (Metrohm, Herisau, Switzerland) The water content was found to be less than 0.03% for the tested ionic liquid, and it was taken into account for further calculations. The purity and the provenance of all samples are given in Table S1.

### 3.2. Determination of the Physicochemical Properties of [Bmim][Man]

Density measurements of the synthesized IL were performed at atmospheric pressure (0.1 MPa) using a Rudolph Research Analytical DDM 2911 vibrating-tube densimeter over the temperature range *T* = (293.15–323.15) K. The instrument accuracy was ±0.00005 g·cm^−3^, with repeatability better than 0.01% and a standard uncertainty < 3·10^−4^ g·cm^−3^. Before each series of measurements, the device was calibrated with triple-distilled water and air at 293.15 K. Temperature control was ensured by the built-in Peltier thermostat, giving a temperature uncertainty under 0.015 K. Viscosity effects were automatically corrected, and each reported density value represents an average of at least five measurements using approximately 1 cm^3^ of sample. The instrument contained an integrated moisture trap.

Viscosity was determined with a Brookfield DV II+ Pro viscometer equipped with a SC4-18 spindle and thermostated to within ±0.01 K. About 15 cm^3^ of pure IL was used for each run. Rotation speeds between 0.2 and 2 RPM were applied to maintain an appropriate torque. The device was calibrated with certified viscosity standards. Each viscosity value corresponds to the mean of three measurements, with a relative standard uncertainty of about 0.02.

Electrical conductivity was measured using a Pyrex cell with platinum electrodes and a Jenco 3107 conductivity meter (DC mode) over the same temperature range *T* = (293.15–323.15 K). The cell was calibrated using 0.1000 mol·dm^−3^ KCl, yielding a cell constant of 1.0353 cm^−1^, which was periodically verified. To avoid electrode heating and polarization, at least ten readings were taken at 5 s intervals, and the three measurement averages are reported. The relative standard uncertainty in conductivity was under 1.5%.

Thermogravimetric analysis (TGA) was performed on a TGA/DSC 1 (Mettler-Toledo) instrument. Samples of approximately 5–10 mg were heated from 25 °C to 400 °C at a constant rate of 10 °C·min^−1^ under a nitrogen atmosphere (flow rate 50 mL·min^−1^). The onset decomposition temperature (*T*ₒₙₛₑₜ) was determined from the intersection of the baseline weight and the tangent to the mass-loss curve.

### 3.3. Viscosity Measurements of [Bmim][Man] Aqueous Solutions

The dynamic viscosity of the aqueous [Bmim][Man] solutions was determined using an Ubbelohde capillary viscometer. Flow times for each solution were measured with a digital stopwatch, which has an accuracy of ±0.01 s. To ensure precision, each sample’s flow time measurement was repeated at least ten times, with an average reproducibility of less than 0.02%. To account for instrument-specific factors, the viscometer was first calibrated. The calibration constants, *L* (0.0206 cm^2^·s^−2^) and *K* (2.5445 cm^2^), were determined by measuring the flow time of pure water at 293.15 K and 298.15 K.

For each experimental run, the kinematic viscosity (*ν*, in cm^2^·s^−1^) of the ionic liquid solution was calculated from the average flow time using the established calibration equation:

(9) $$\nu =L\cdot t-\frac{K}{t}$$

where *t* (s) is the flow time, *L* and *K* are the instrument constants. The dynamic viscosity (*η*, in mPa·s) was subsequently derived by multiplying the kinematic viscosity by the density (*d*, in g·cm^−3^) of the corresponding solution, as shown in the following relation:

*η* = *ν*⋅*d* (10)

Throughout all measurements, the temperature of the sample cell was strictly controlled within ±0.01 K of the setpoint, with an overall standard uncertainty of ±0.015 K for the controlled temperature.

### 3.4. Determination of the Antimicrobial Activity

The antimicrobial activity of [Bmim][Man], its precursor salt [Bmim]Cl, and sodium mandelate (NaMan) was assessed using the broth microdilution method following adapted CLSI/EUCAST recommendations. The tested organisms included *Escherichia coli* (ATCC 25922), *Pseudomonas aeruginosa* (ATCC 27853), and the filamentous fungi *Penicillium verrucosum*, *Aspergillus flavus*, and *Aspergillus parasiticus*, obtained from the culture collection of the Microbiology Laboratory, University of Novi Sad.

*E. coli* (ATCC 25922) and *P. aeruginosa* (ATCC 27853) are standard, well-characterized Gram-negative bacterial strains recommended by CLSI/EUCAST for antimicrobial susceptibility testing. They represent models for opportunistic pathogens with different membrane characteristics and are commonly used to evaluate the activity of new antimicrobial agents, including ionic liquids. The chosen filamentous fungi (*P. verrucosum, A. flavus*, and *A. parasiticus*) are relevant from both environmental and mycotoxigenic perspectives. *A. flavus* and *A. parasiticus* are producers of carcinogenic aflatoxins, making them significant in food safety and public health. Testing against these fungi provides initial data on the potential antifungal utility of the IL beyond antibacterial effects.

Bacterial suspensions were adjusted to the turbidity of a 0.5 McFarland standard (≈1–2·10^8^ CFU·ml^−1^), while fungal spore suspensions (≥10^6^ spores·mL^−1^) were prepared in saline. For the assays, inoculated Mueller-Hinton or Sabouraud Dextrose broth was diluted into 96-well microplates so that the final inoculum reached approximately 1–2·10^6^ CFU·mL^−1^ for bacteria and 1-4·10^6^ spores·mL^−1^ for fungi. Two-fold serial dilutions of the tested compounds covered a concentration range corresponding to that applied in the original procedure (approx. 3.5–900 mmol).

Microplates were incubated for 24 h at 37 °C (bacteria) or 72 h at 25–26 °C (fungi). The MIC was defined as the lowest concentration without visible growth compared with the inoculated control. To determine MBC/MFC values, aliquots from wells showing no growth were transferred to Nutrient agar or Malt Extract agar and incubated under the same temperature and time conditions. The lowest concentration producing ≥ 99.9% reduction in viable cells or spores was recorded as the MBC or MFC. All antimicrobial assays were performed in triplicate, and the reported MIC/MBC/MFC values represent the median of three independent experiments.

### 3.5. ABS Preparation and Extraction Procedure

**Figure 11:**
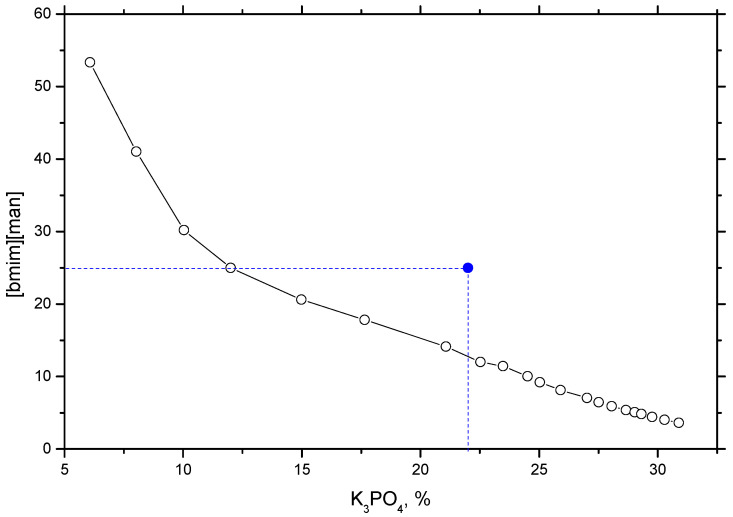
Ternary phase diagrams of the studied ABS {[Bmim][Man] + K_3_PO_4_ + H_2_O} at *T* = 296 K and atmospheric pressure (*p* = 0.1 MPa). ● extraction point.

To determine the extraction point, a ternary phase diagram for the ABS {[Bmim][Man] + K_3_PO_4_ + H_2_O} was determined experimentally using the cloud point titration method at (293 ± 1) K and atmospheric pressure (*p* = 0.1 MPa) [21]. Briefly, an aqueous K_3_PO_4_ solution (ω = 40%) was added dropwise to an aqueous IL solution (ω = 62%) until the onset of turbidity. Deionized water was then added until the system became clear again. These two steps were repeated until no further turbidity was observed. The masses of all added solutions were measured on an analytical balance (CP224S, Sartorius, accuracy of ±10^−4^ g). After each addition step, the mixture was homogenized using a vortex mixer (Fisher Scientific, Pittsburgh, Pennsylvania) at 2500 rpm. The resulting ternary phase diagram for the {[Bmim][Man] + K_3_PO_4_ + H_2_O} system, together with the extraction point, is presented in Figure 11. The region above the curve represents the two-phase area, where the system separates into an IL-rich top phase and a salt-rich bottom phase.

For the extraction studies, aqueous stock solutions of lanthanide ions (La^3+^, Ce^3+^, Nd^3+^, and Dy^3+^), In^3+^, and transition metal ions (Ni^2+^ and Cu^2+^) were prepared from the corresponding nitrate salts at an initial concentration of 1 g L^−1^ for each metal. A multicomponent aqueous solution containing all studied metal ions was then prepared, with the concentration of each ion adjusted to 100 mg L^−1^. Based on the binodal curve (Figure 11), the extraction point was selected at 25% [Bmim][Man], 22% K_3_PO_4_, and 53% multicomponent aqueous solution. The total mass of ABS was 1.00 g. The ABS was vigorously vortexed and then allowed to equilibrate at 296 K for 1 h. The phases were carefully separated, and the concentration of metal ions in each phase was quantified by inductively coupled plasma optical emission spectrometry (ICP-OES, Thermo Scientific). The partition coefficient (*K*) for each metal was calculated as *K* = *c*_IL-rich_/*c*_salt-rich_, where *c* is the equilibrium concentration of the metal in the respective phase. The extraction efficiency (%E) for each metal into the IL-rich phase was calculated as %E = (*m*_IL-rich_/*m*_in_) × 100%, where *m*_IL/rich_ is the mass of the metal in the IL-rich phase and *m*_in_ is the total mass of metal initially added to the system.

## 4. Conclusions

This study successfully synthesized and comprehensively characterized the ionic liquid [Bmim][Man], demonstrating that the strategic introduction of a hydroxyl group onto the aromatic mandelate anion is a powerful design strategy for creating multifunctional ILs. The key conclusions are as follows:

- The mandelate anion, with its aromatic ring, carboxylate, and hydroxyl group, imposes a profound influence on the IL’s properties. [Bmim][Man] exhibits the highest density and viscosity in its pure state among the compared ILs, a direct consequence of strong, directional hydrogen bonding leading to tight ion packing and restricted mobility. The high molar conductivity despite high viscosity suggests that these same H-bonding interactions may also promote a degree of ion dissociation, increasing the number of charge carriers.
- [Bmim][Man] demonstrated the highest thermal stability (*T*ₒₙₛₑₜ = 232.2 °C) in the series. This is attributed to the stabilizing effect of the mandelate anion, likely through a combination of electron delocalization across the aromatic system and intramolecular hydrogen bonding, which enhances its resistance to decomposition.
- The IL exhibits a strong tendency to self-aggregate in aqueous solution, as evidenced by the lowest Critical Aggregation Concentration (CAC = 0.01982 mol·dm^−3^) among its peers. This behavior is driven by the synergistic effect of π-π stacking between the aromatic rings and extensive intermolecular hydrogen bonding involving the hydroxyl and carboxylate groups.
- [Bmim][Man] displays significantly enhanced antibacterial activity against *E. coli* and *P. aeruginosa* compared to its individual ionic components ([Bmim][Cl] and NaMan), indicating a synergistic effect. It also shows moderate, specific antifungal activity against *A. parasiticus*. This makes it a promising candidate for applications requiring dual functionality as a tunable solvent and a bioactive agent.
- The preliminary results on metal extraction using [Bmim][Man]-based ABS show a selective partitioning behavior for different transition metals, highlighting its potential as a designer solvent in separation processes, which is directly correlated to the complexation strength of these metals with the mandelate anion.

In summary, the strategic introduction of the hydroxyl-functionalized mandelate anion into the [Bmim]^+^ framework has yielded a multifunctional ionic liquid with a unique combination of strong intermolecular interactions, high thermal stability, low aggregation threshold, and enhanced biological activity. The demonstrated multifunctionality paves the way for its application in areas such as antimicrobial coatings, green hydrometallurgy for selective metal recovery, and as a functional component in smart materials. Future work will investigate its cytotoxicity and biodegradability to assess its green credentials fully.

## Figures and Tables

**Table 1:** MIC and MBC/MFC values (mmol·L^−1^) of tested IL and its individual cationic and anionic components against bacteria and filamentous fungi.

	Bacteria				Fungi					
	*E. coli*		*P. aeruginosa*		*P. verrucosum*		*A. flavus*		*A. parasiticus*	
*c* (mmol·L^−1^)	MIC	MBC	MIC	MBC	MIC	MFC	MIC	MFC	MIC	MFC
[Bmim][Cl]	56.3	112.6	112.6	450.4	900	900	>900	900	>900	900
NaMan	450.4	450.4	450.4	450.4	900	900	>900	>900	>900	>900
[Bmim][Man]	28.1	112.6	14.1	56.3	900	hell900	>900	>900	450.4	450.4

## Data Availability

Data are available from the authors if required.

## Supplementary Materials

The following supporting information can be downloaded at: https://www.mdpi.com/article/10.3390/molecules30244824/s1. Table S1: Provenance and purity of the samples; Table S2: Density, electrical conductivity, molar conductivity, and viscosity values of [Bmim][Man]; Table S3: Molalities (*m*) and calculated dynamic viscosities (*η*) for the studied aqueous ionic liquid solutions; Figure S1: ^1^H and ^13^C NMR spectra of [Bmim][Man]; Figure S2: FTIR spectra of [Bmim][Man].
