# Supplementary material for: 1-Butyl-3-methylimidazolium Mandelate: A Multifunctional Ionic Liquid with Enhanced Hydrogen Bonding, Thermal Stability, Antimicrobial Activity, and Extraction Capability
Source: Molecules. 2025 Dec 18;30(24):4824. doi: 10.3390/molecules30244824 (PMC12735414; doi:10.3390/molecules30244824)
Supplement: Supplementary file 1 [file molecules-30-04824-s001.zip › molecules-4044481-supplementary.pdf]

Supplementary file

1-Butyl-3-methylimidazolium Mandelate: A Multifunctional Ionic Liquid with Enhanced Hydrogen Bonding, Thermal Stability, Antimicrobial Activity, and Extraction Capability

Nikolett Cakó Bagány[1], Eleonora Čapelja[1], Sanja Belić[1], Dajana Lazarević[2], Jelena Jovanović[2], Tatjana Trtić-Petrović[2], Slobodan Gadžurić[1*]

[1]*Faculty of Science, University of Novi Sad, Trg Dositeja Obradovića 3, 21000 Novi Sad, Serbia*
[2]*Laboratory of Physics, Vinča Institute of Nuclear Sciences – National Institute of the Republic of Serbia, University of Belgrade, Mike Petrovića Alasa 12-14, 11001 Belgrade, Serbia*

[*] Corresponding author: e-mail: slobodan.gadzuric@dh.uns.ac.rs; Tel: +381 21 485 2744 Fax: +381 21 454 065

Table S1. Provenance and purity of the samples

Chemical name	Source	Mass fraction purity	Purification method
1-butyl-3-methylimidazolium chloride	Sigma Aldrich	98%**	None
Mandelic acid	Sigma Aldrich	99%**	None
Methanol	J.T. Baker	> 99%**	None
1-butyl-3-methyimidazolium mandelate	Synthesis	≥ 98%*	Rotary evaporation followed by vacuum

*Determined by NMR measurements.
**Provided by the supplier.

Table S2. Density, electrical conductivity, molar conductivity, and viscosity values of [Bmim][Man]

T (K)	d (g·cm^{-3})	κ (mS·cm^{-1})	Λ_m (S·cm^2·mol^{-1})	η (mPa·s)
293.15	1.13552	0.127	0.033	1462.46
298.15	1.13215	0.218	0.056	952.61
303.15	1.12872	0.366	0.094	672.44
308.15	1.12524	0.512	0.132	502.98
313.15	1.12163	0.715	0.185	395.69
318.15	1.11798	0.949	0.246	320.56
323.15	1.11426	1.377	0.358	265.53

Table S3. Molalities (m) and calculated dynamic viscosities (η) for the studied aqueous ionic liquid solutions

m (mol·kg^{-1})	T (K)				
	293.15	298.15	303.15	308.15	313.15
	η (mPa·s)				
[Bmim][Man]					
0.01001	1.009	0.896	0.801	0.719	0.647
0.01501	1.012	0.898	0.803	0.721	0.654
0.02006	1.015	0.901	0.807	0.725	0.656
0.03000	1.024	0.909	0.812	0.731	0.660
0.04006	1.033	0.919	0.819	0.738	0.665
0.05028	1.043	0.928	0.827	0.744	0.670
0.07055	1.067	0.947	0.844	0.756	0.684
0.10025	1.105	0.974	0.864	0.775	0.699

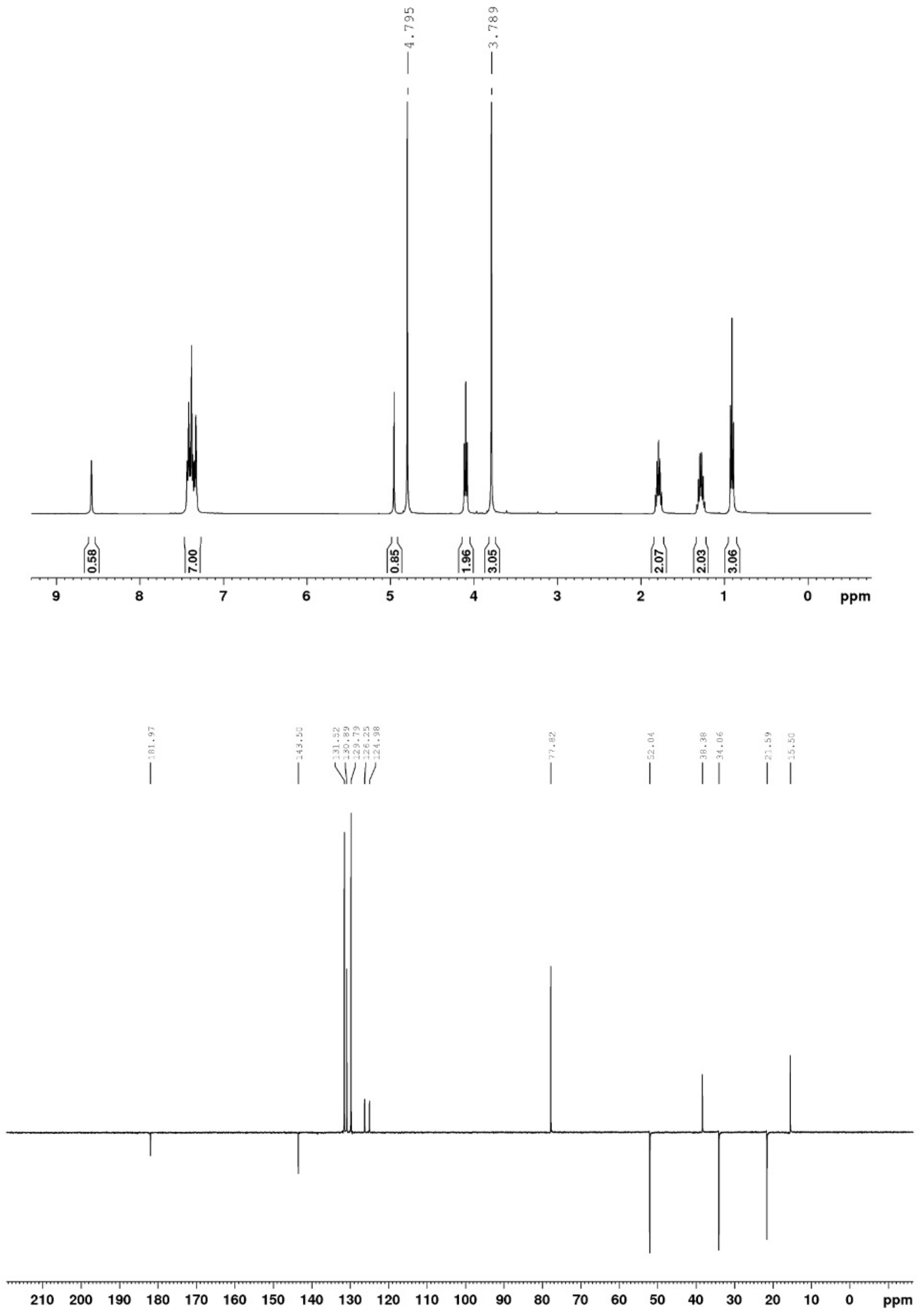

Figure S1. ^{1}H and ^{13}C NMR spectra of [Bmim][Man]

^{1}H NMR (D_2O): 0.91, (t, 3H, *J*= 7.6 Hz, $NCH_2CH_2CH_2C\mathit{H}_3$); 1.28, (m, 2H, $NCH_2CH_2C\mathit{H}_2CH_3$); 1.79, (m, 2H, $NCH_2C\mathit{H}_2CH_2CH_3$); 3.79, (s, 3H, $NC\mathit{H}_3$); 4.09, (t, 2H, *J*= 7.3 Hz, $NC\mathit{H}_2CH_2CH_2CH_3$); 4.96, (s, 1H, C*H*OH); 7.25-7.53, (m, 7H, H-4, H-5 i Ar-H); 9.7, (s, 1H, H-2).

^{13}C NMR (D_2O): 15.50 ($NCH_2CH_2CH_2\mathit{C}H_3$); 21.59 ($NCH_2CH_2\mathit{C}H_2CH_3$); 34.06 ($NCH_2\mathit{C}H_2CH_2CH_3$); 38.38 ($N\mathit{C}H_3$); 52.04 ($N\mathit{C}H_2CH_2CH_2CH_3$); 77.82 (*C*HOH); 124.98 (C-5); 126.25 (C-4); 129.79 (C-3' i C-5'); 130.89 (C-4'); 131.52 (C-2' i C-6'); 138.49 (C-2); 143.50 (C-1'); 181.97 (C=O).

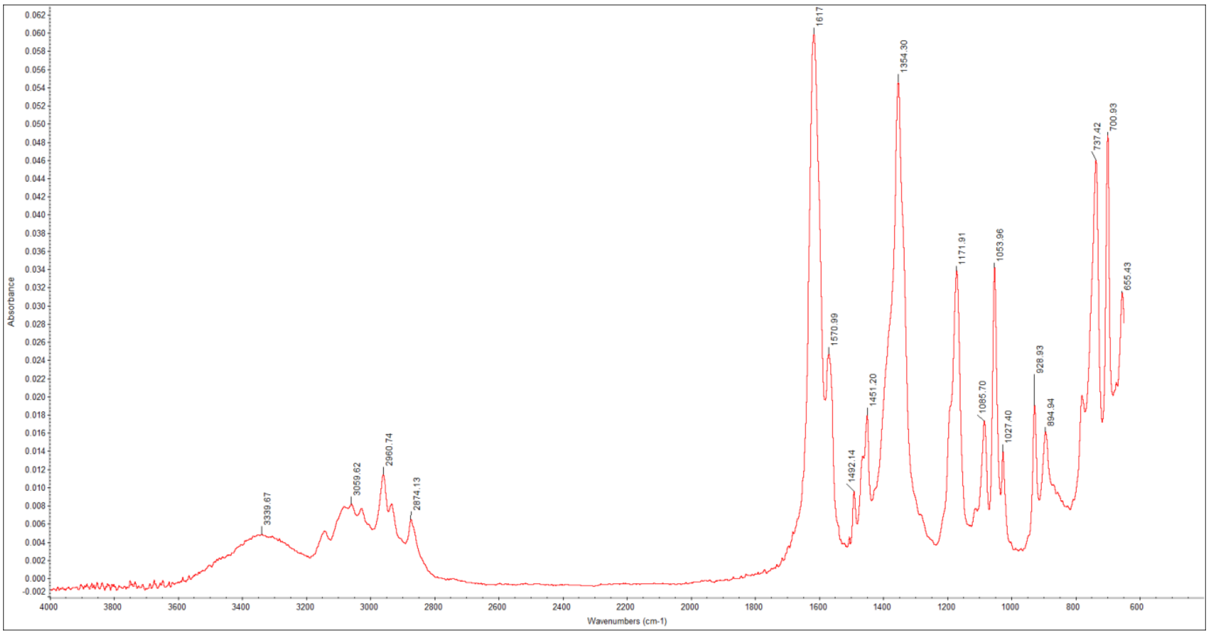

Figure S2. FTIR spectra of [Bmim][Man]

IR (neat): 3339 (stretching vibration of –OH group); 2960 (sym. stretching vibration $–CH_3$ group bound to N); 1570 (stretching vibration of the acetate ion); 1354 (rocking vibration H-C-H of butyl group); 1171 (bending vibration of imidazolium ring); 737 (in-plane bending vibration of monosubstituted aromatic ring).
